# The Modified Coffee Cup Model: A Novel Approach to Teaching Cavernous Sinus Anatomy

**DOI:** 10.7759/cureus.82562

**Published:** 2025-04-19

**Authors:** Praisy Joy, Siva Priya, Kanneeswaran Loganathan, Arunkumar Sekar, Sipra Rout, Manisha R Gaikwad, Arthi Ganapathy

**Affiliations:** 1 Anatomy, All India Institute of Medical Sciences, Bhubaneswar, IND; 2 Neurosurgery, All India Institute of Medical Sciences, Bhubaneswar, IND; 3 Anatomy, All India Institute of Medical Sciences, New Delhi, IND

**Keywords:** cavernous sinus (cs), medical school education, peer-assisted learning, team-based learning (tbl), vertical integration

## Abstract

Background and aim

The cavernous sinus has a complex anatomy, containing numerous neurovascular structures arranged in intricate spatial relationships. Teaching its anatomy is often limited to didactic lectures, which make it challenging for students to visualize its 3D configuration. The "coffee cup model" offers a hands-on approach to improve understanding of the neurovascular structures within the cavernous sinus and its surrounding triangles, enhancing psychomotor skills and comprehension. This study aimed to help undergraduate MBBS students grasp the 3D anatomy of the cavernous sinus using the coffee cup model. Additionally, it sought to reinforce learning through vertically integrated lectures combining anatomy and neurosurgery.

Materials and methods

This study involved students reviewing the cavernous sinus anatomy beforehand. A pre-test with questions on this topic was followed by a combined lecture from an anatomist and a neurosurgeon. Students then participated in a group activity, constructing the coffee cup model. Post-test scores were compared with pre-test results using the Student’s t-test, and feedback was collected.

Results

Students showed enthusiasm during the activity, creating innovative models that reflected an improved understanding of the cavernous sinus anatomy. Post-test performance significantly exceeded pre-test results, demonstrating the model's effectiveness.

Conclusion

The coffee cup model, integrated with neurosurgical perspectives, enhanced students’ understanding of the 3D anatomy of the cavernous sinus, making it an effective and interactive teaching method.

## Introduction

Traditionally, the teaching of anatomy has relied heavily on lectures, textbooks, cadaveric dissections, and demonstrations from prosected specimens. However, as medical education evolves, there is a growing recognition of the need for more interactive and experiential learning methods. In response to this, universities have been advocating for curriculum reforms aimed at moving away from passive learning approaches towards more proactive and engaging formats, including problem-based learning (PBL), early clinical exposure, computer-assisted learning, and physical models [1].

Physical models of the human body have long been utilized in anatomy education to provide students with a tangible representation of anatomical structures. These models offer a means to bridge theoretical knowledge with practical application, allowing for a more comprehensive understanding of complex anatomical relationships. Despite variations in their degree of resemblance to the human body, physical models remain valuable tools in anatomical education, offering students the opportunity to visualize and manipulate anatomical structures in three-dimensional space [2,3].

In addition to the utilization of physical models, the implementation of vertical integration within a specific teaching-learning module has been demonstrated to be highly effective in enhancing understanding of the subject matter. Vertical integration has emerged as another important aspect of modern medical education, emphasizing the integration of basic science concepts with clinical practice across the curriculum. By gradually decreasing lecture-based learning and increasing exposure to clinical settings over time, vertical integration aims to facilitate meaningful learning experiences that align with the way knowledge is applied in clinical practice. This approach not only enhances memory retrieval but also fosters the development of relevant knowledge frameworks, ultimately better preparing students for the challenges of clinical practice [4,5].

We hypothesize that using a modified coffee cup model for teaching cavernous sinus anatomy, along with vertical integration of clinical aspects, will result in a significant improvement in undergraduate medical students' understanding of the anatomy and clinical relevance of the cavernous sinus compared to traditional didactic lecture-based learning methods. Additionally, this approach will enhance students' psychomotor skills related to anatomical representation.

## Materials and methods

Study setting

The study conducted at All India Institute of Medical Sciences (AIIMS), Bhubaneswar, highlights an innovative approach to teaching anatomy to first-year MBBS students by integrating traditional lectures with hands-on activities and vertical integration of gross and clinical anatomy. The focus of this study was the cavernous sinus, a complex anatomical structure with significant clinical relevance. A combination of pre-test/post-test evaluations and interactive 3D modeling activities was used to assess and enhance students’ understanding of this topic.

Study design

The study recruited undergraduate students from the 2023-24 MBBS batch, ensuring ethical standards through committee approval and informed consent. Out of 106 initial participants, 104 completed the study. The design was observational and questionnaire-based, structured to evaluate the students' learning outcomes before and after the teaching intervention. A pre-test was conducted using Google Forms (Menlo Park, CA: Google LLC) to assess their baseline knowledge. The questionnaire comprised the following four sections: consent, demographic details, six questions on the gross anatomy of the cavernous sinus, six questions on its clinical anatomy, and feedback on the activity in the post-test phase.

The methodology was meticulously planned to maximize engagement and learning. Students were instructed to study the anatomy of the cavernous sinus independently prior to the intervention. The lectures were delivered in two parts as follows: the first, by a faculty member referred to as PJ, focused on the gross anatomy of the cavernous sinus, and the second, by AS, covered its clinical relevance. This sequential structure facilitated vertical integration, linking anatomical knowledge to its clinical applications, a method increasingly recognized as crucial in medical education.

Following the lectures, students participated in a two-hour hands-on activity aimed at reinforcing their understanding. Divided into 25 groups, they were tasked with constructing a 3D model of the cavernous sinus using paper coffee cups and colored pipe cleaners. This exercise encouraged teamwork, creativity, and the application of theoretical knowledge in a practical context. Groups presented their models to faculty members, allowing for interactive discussions and immediate feedback.

Analysis

The post-test, also conducted via Google Forms, measured the improvement in students’ knowledge following the intervention. Feedback was collected to evaluate the students' perceptions of the teaching method. The data from pre- and post-tests, as well as feedback responses, were analyzed using Microsoft Excel (Redmond, WA: Microsoft Corp.). Improvements in test scores were calculated to quantify the effectiveness of the teaching intervention.

The study’s design effectively incorporated active learning techniques, which have been shown to improve retention and understanding of complex subjects like anatomy. The use of 3D modeling allowed students to visualize the spatial relationships within the cavernous sinus, addressing the challenges of learning this intricate structure through lectures alone. Furthermore, vertical integration bridged the gap between theoretical knowledge and clinical relevance, underscoring the practical importance of anatomical education.

The incorporation of feedback demonstrated a commitment to continuous improvement in teaching methods. Providing chocolates as incentives helped maintain a positive and motivating environment for students. This study exemplifies how innovative, interactive, and student-centered teaching approaches can significantly enhance the learning experience in medical education. By fostering engagement, collaboration, and application of knowledge, such methods can be instrumental in preparing students for clinical practice.

## Results

Pre-test

Out of 125 students, 106 students had given consent and answered the pre-test. The student participants were in the age group between 17 and 22 years. Twenty-two participants were females and 84 were males. The questions asked to assess the knowledge of the students before the conduct of this exercise were as in Table 1.

**Table 1:** Questions asked for pre-test and post-test during the activity. The first six questions were on the gross anatomy of CS. The last six questions were on clinical anatomy of CS. CVT: cavernous venous thrombosis; CS: cavernous sinus

S. no	Questions of CS	Percentage of correct answers in pre-test	Percentage of correct answers in post-test	Remarks
1	CS is situated in which fossa	95.3%	99%	3.7% increase
2	Abducens nerve forms which relation to CS	81.1%	85.6%	4.5% increase
3	Structure that is not related to lateral wall of CS	96.2%	100%	3.8% increase
4	Content of Dorello’s canal	91.5%	96.2%	5.3% increase
5	Posterior wall of CS is made up of	49.1%	58.7%	9.6% increase
6	CS is developed	68.9%	99%	30.1% increase
7	Jacod's triad in cavernous sinus thrombosis does not involve	84.9%	89.4%	4.5% increase
8	Carotid-cavernous fistula is formed between	90.6%	97.1%	6.5% increase
9	Spread of infection to CS is by	82.1%	90.4%	8.3% increase
10	CVT is mostly commonly due to	81.1%	88.5%	7.4% increase
11	The clinical procedure depicted below helps to evaluate the clinical sign presented in	82.1%	93.3%	11.2% increase
12	One of the signs of carotico-cavernous fistula is	65.1%	76.1%	11% increase

Gross Questions

A total of 101 (95.3%) participants answered that the cavernous sinus was located in the middle cranial fossa. Eighty-six (81.1%) participants answered correctly that the abducens nerve was related laterally to the internal carotid artery. In total, 102 (96.2%) participants answered the third question correctly, and 97 (91.5%) participants answered the fourth question correctly. To the fifth question on the posterior wall of the cavernous sinus, 52 (49.1%) students answered correctly. Seventy-three (68.9%)students answered that the cavernous sinus is formed before birth (Table 1).

Clinical Questions

Nienty (84.9%) participants knew about the Jacod’s triad, and 96 (90.6%) participants knew how the carotico-cavernous fistula is formed. Eighty-seven (82.1%) student participants answered correctly that the spread of infection to cavernous sinus is by the facial vein, and 86 (81.1%) participants answered that bacterial infection was the most common infection of the cavernous sinus. The last two questions were related to a picture of carotico-cavernous fistula. Eighty-seven (82.1%) participants answered that the picture question was carotico-cavernous fistula. Sixty-nine (65.1%) participants answered that pulsatile proptosis was one of the classical clinical signs of carotico-cavernous sinus fistula (Table 1).

Activity

**Figure 1:**
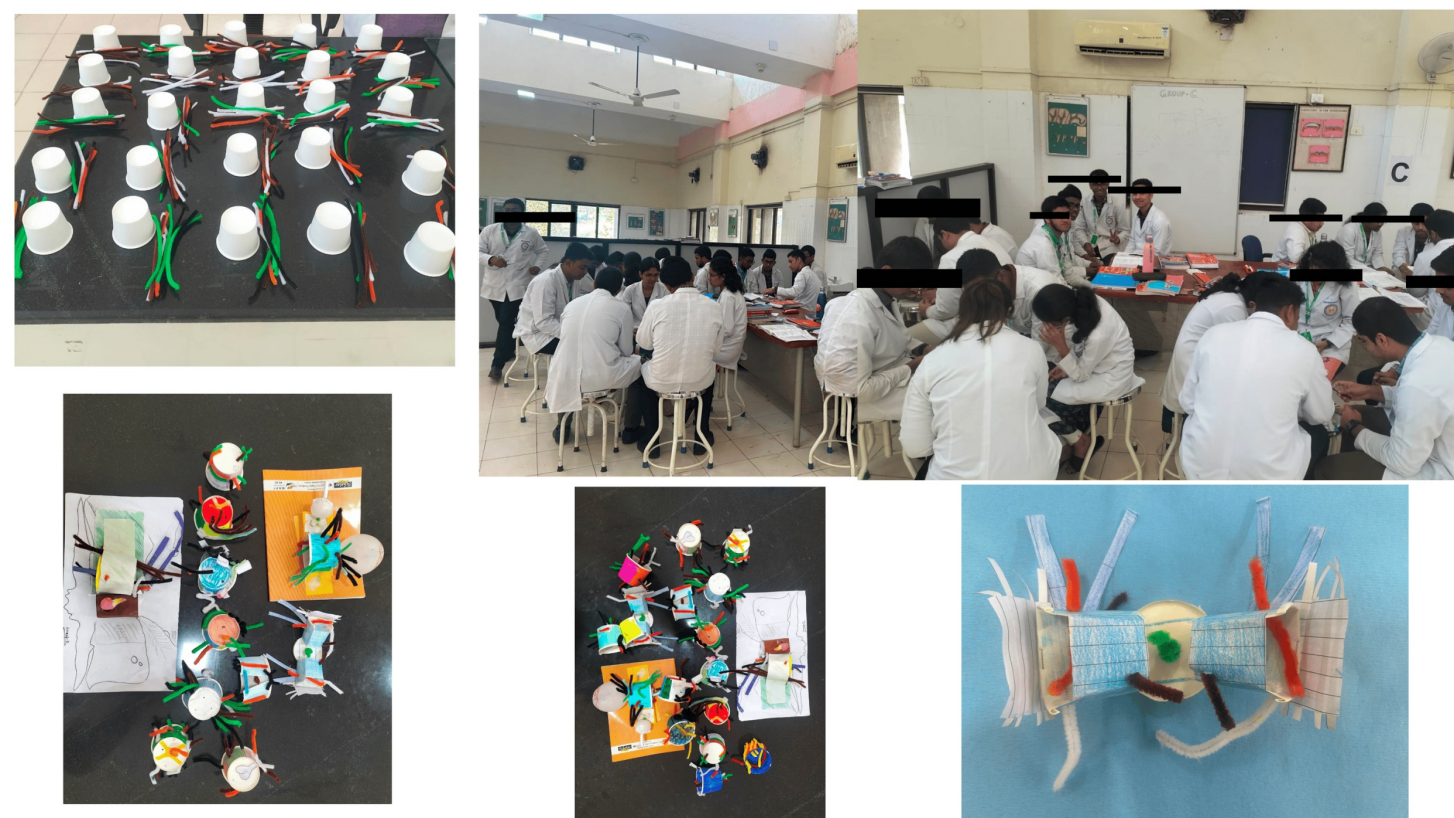
The models made by students during the activity. Team-based learning is described in these pictures. The students have consented to be photographed.

A lecture was given on the anatomy and clinical aspects of the cavernous sinus. The duration of the lecture was 40 minutes. The anatomical aspect was covered by PJ, and the clinical aspects of the cavernous sinus were covered by AS. The lecture was delivered using PowerPoint, and a video clip of a cavernous sinus thrombosis case was shown to the students. After 1 hour of lecture, the students were divided into five groups, and each group had 25 students. Then the five bigger groups were divided into five smaller groups, each group containing five students. The allotment was random. The group activity given was that the students in each group were asked to make a 3D model of cavernous sinus. A coffee cup and eight coloured pipeliners were given to the students. At the end of one and a half hours, the students were asked to present their model to the group of teachers. The students made some amazing models (Figure 1).

Their concepts regarding the cavernous sinus were very clear. Reinforcing the concepts by small group teaching was done to clarify the concept at the end of the activity.

Post-test

A total of 104 responses were recorded in the post-test Google Form.

Gross Questions

A total of 103 (99%) student participants answered the first question correctly. The relationship of abducens nerve to the internal carotid artery (ICA) was answered correctly by 89 (85.6%) students. A total of 104 (100%) answers were correct regarding the structures on the lateral wall of cavernous sinus. Hundred (96.2%) students answered correctly regarding the Dorello’s canal. Sixty-one (58.7%) students answered correctly regarding the posterior wall of the cavernous sinus. In total, 103 (99%) answered correctly that the cavernous sinus is formed before birth.

Clinical Questions

Ninety-three (89.4%) students knew about Jacod’s triad. A total of 101 (97.1%) students answered the second question correctly. Ninety-four (90.4%) students answered the question on the spread of infection to cavernous sinus correctly. Ninety-two (88.5%) students answered that bacterial infection was the most common in cavernous sinus. Ninety-seven (93.3%) students answered the fifth question correctly, and 79 (76%) students answered the sixth question correctly.

Comparison of pre-test and post-test performance

There was a significant increase in the correct responses of the students in the post-test performance after the activity. There was an average 8.82% increase in the student performance when pre- and post-test performance were compared. There was an average of 9.5% increase in the gross component and an average of 8.15% increase in the clinical component at the end of the activity.

Statistics

The pre-test and post-test marks were entered into an Excel sheet, and a paired t-test was performed. Except for questions 1, 2, 4, and 5, the other questions showed statistically significant results when compared (Table 2). This can be seen in the table in the appendix.

**Table 2:** The comparison between pre-test and post-test average scores by paired t-test. Except for questions 1, 2, and 4, all questions show statistical significance. Notably, question 6 had a very low p-value. Q: question

Q. no.	Pre-test average scores	Post-test average scores	p-Value
Q1	4.78	4.94	0.08
Q2	4.08	4.35	0.16
Q3	4.78	5.00	0.04
Q4	4.62	4.78	0.25
Q5	2.36	2.90	0.06
Q6	3.49	5.00	1.03082E-08
Q7	4.30	4.56	0.05
Q8	4.46	4.83	0.007
Q9	4.08	4.56	0.002
Q10	3.97	4.35	0.03
Q11	4.08	4.73	0.001
Q12	3.22	3.81	0.01

Feedback

**Figure 2:**
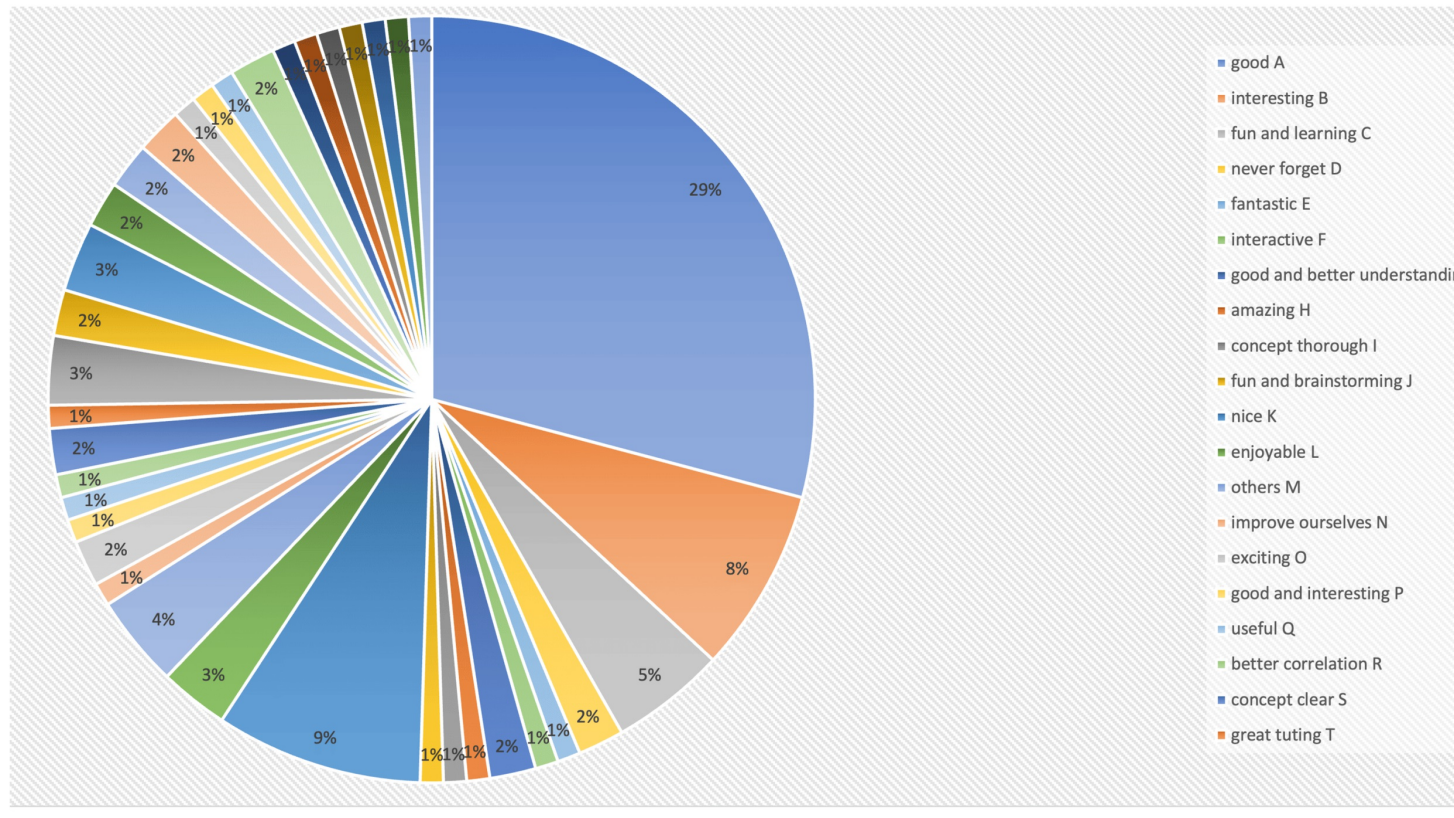
The themes that were received as feedback from students. When asked about their experience, students gave very positive feedback about the exercise. "Good," "interesting," "fun," "educational," and "unforgettable" were among the most common responses.

**Figure 3:**
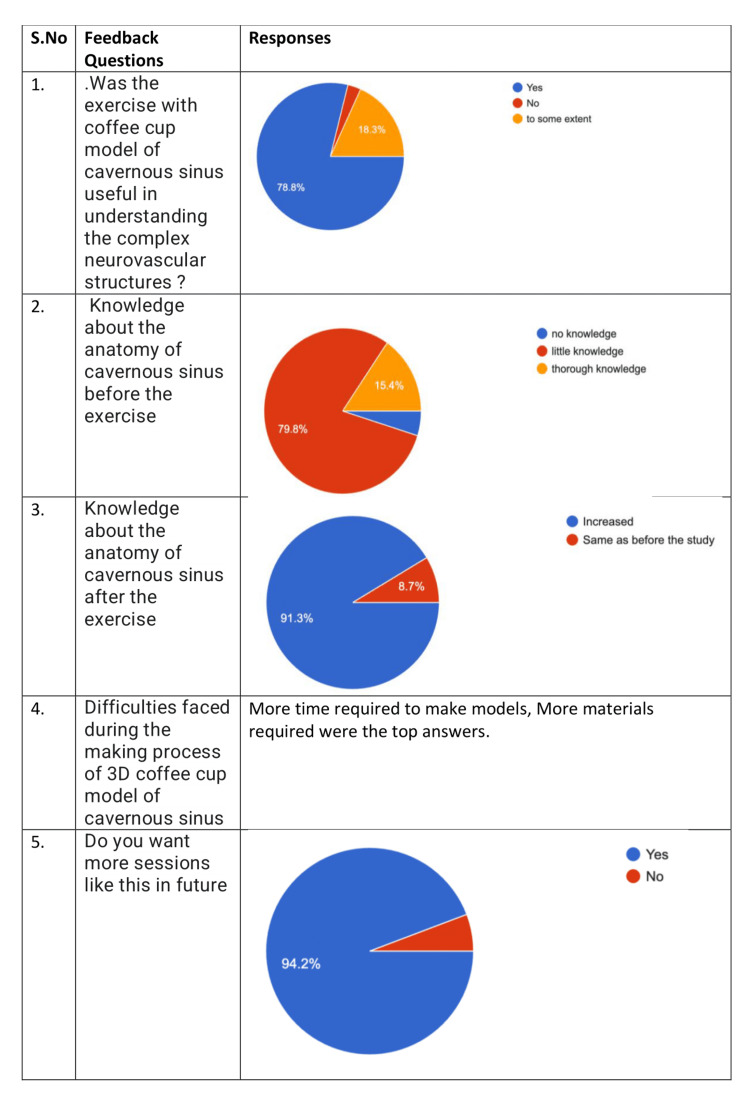
Questions one to five regarding feedback and the overall responses of the students.

**Figure 4:**
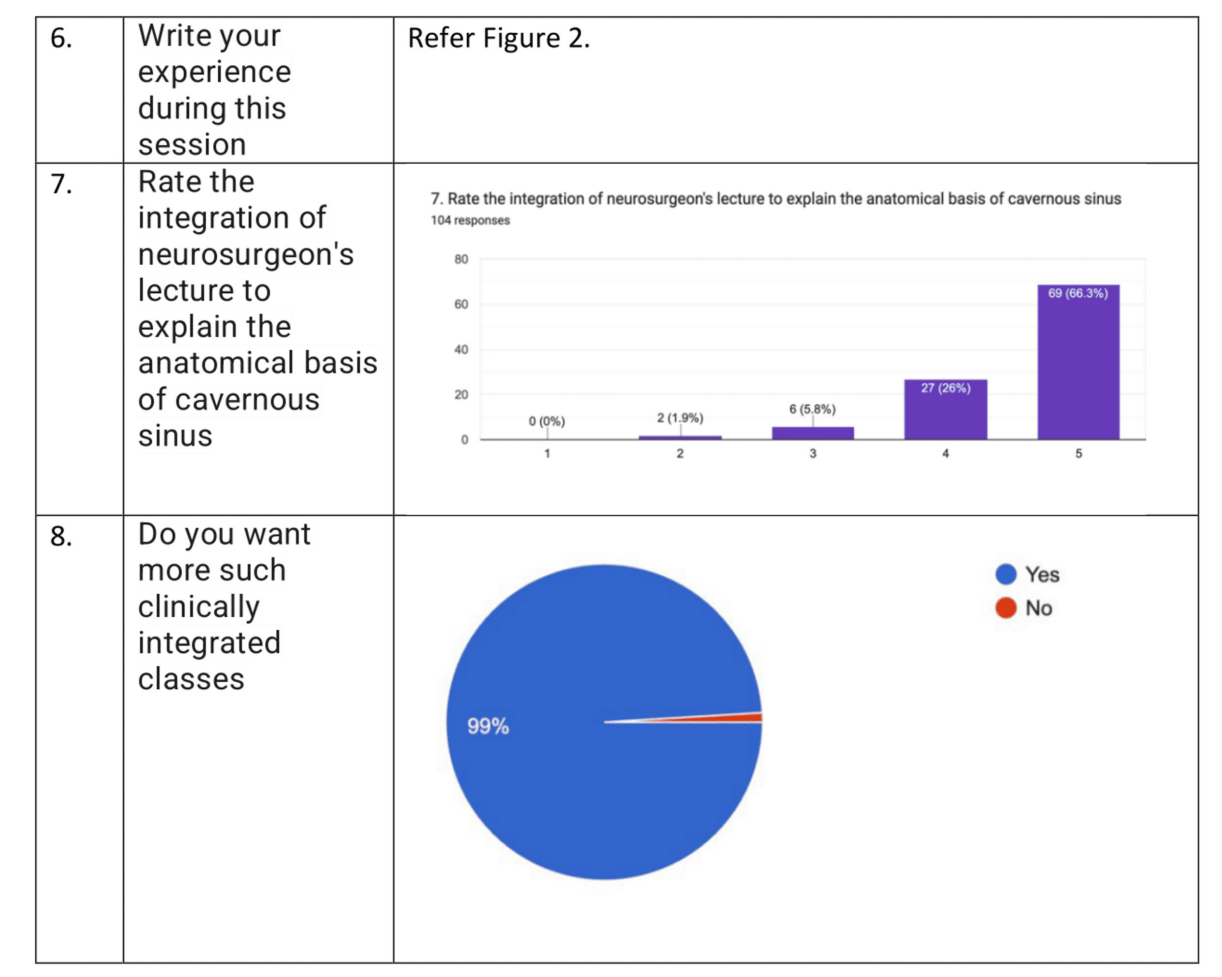
Feedback questions six to eight. Questions six to eight reflect the feedback responses given by students. Question seven responses are given on a Likert scale.

When asked about the experience, the students have written that it was “fun,” “amazing,” and “interesting” exercise. It helped them understand the concept better. It sparked their interest in reading anatomy. It made their concept thorough. On the whole, it was an enjoyable exercise and a good initiative. This is represented in Figure 2. When asked about the difficulty they faced, most of the students wrote about the lack of materials, time management, and orientation of the structures in making the model. The students gave positive feedback for the activity. When asked if the whole activity was helpful in understanding the complex neurovascular structures, 78.8% (82) of the students answered yes, 18.3% (19) of the participants answered to some extent, and 2.9% (3) of the students felt that the activity did not increase their knowledge. When asked about the knowledge before the study, 79.8% (83) of the students answered that there was minimal knowledge before the study, 4.8% (5) of the students answered that there was no knowledge of cavernous sinus (CS) before the study and 15.4% (16) of the students answered they had thorough knowledge before the study. When questioned about the knowledge after the study, 91.3% (95) agreed that the exercise helped in increasing the knowledge of CS, and 8.7% (9) felt that the knowledge remained the same after the exercise. When asked if they wanted more such activities, 94.2% (98) answered yes and 5.8% (6) answered no (Figure 3). When asked to rate on Likert scale about the integration with neurosurgery to explain the clinical aspects, 69 students had given the score of 5, 27 students gave the score of 4, six students gave the score of 3, and two students gave the score of 2.103 (99%) students felt that they wanted more clinically integrated classes (Figure 4).

## Discussion

The modern-day anatomy classroom and students require innovative teaching and learning methods. In modern anatomy teaching, students rely on a variety of resources to enhance their learning experience and deepen their understanding of human anatomy.

The modern anatomy teaching relies on a combination of traditional and innovative resources to provide students with a comprehensive understanding of human anatomy. By integrating a variety of learning modalities, instructors can cater to diverse learning styles and preferences, ensuring that students have access to the tools and resources they need to succeed in their anatomical studies.

Within the scope of anatomical study, the cavernous sinus (CS) presents a particularly complex and clinically significant topic. The CS is a venous space housing vital neurovascular structures and is susceptible to various pathological processes [6,7]. Given its complex anatomy and clinical relevance, the CS provides an ideal candidate for vertical integration within medical education, with input from the faculty of neurosurgery. In this context, the present study aimed to explore the effectiveness of a modified coffee cup model in enhancing the understanding of cavernous sinus anatomy among undergraduate medical students. Originating from Kranzler, the coffee cup model offers a unique hands-on approach to visualizing the neurovascular structures within and around the cavernous sinus. It has been used as a small-group teaching method for neurosurgery residents. By engaging students in the construction of three-dimensional models using simple materials such as coffee cups and colored woolen threads, this model seeks to develop not only students' cognitive understanding but also their psychomotor skills related to anatomical representation [8].

Building upon prior research demonstrating the efficacy of the coffee cup model in enhancing surgical residents' understanding of cavernous sinus anatomy, as well as the benefits of vertical integration, the present study aimed to evaluate the impact of the modified coffee cup model on students' knowledge acquisition and retention.

The modified coffee cup model is a versatile, low-cost, innovative model for teaching cavernous sinus to the students. The students were involved actively, and this model enhanced their active learning. It brought about the novelties described below.

Better psychomotor skills (learning by doing)

Psychomotor skills are critical for medical professionals, as they involve the integration of physical movements with cognitive functions to perform tasks with precision. In medical education, developing psychomotor skills is crucial for clinical competency, especially in areas such as surgical procedures, diagnostic techniques, and patient interaction. A "learning by doing" approach, also known as experiential learning, is one of the most effective ways to foster these skills. Learning by doing emphasizes hands-on practice where learners actively engage with tasks to acquire skills and reinforce knowledge. It encourages deeper learning, better retention, and skill refinement through repetitive practice and feedback. Kaufman and Mann explored the theoretical underpinnings of learning by doing and how it contributed to skill acquisition [9].

Team-based learning (TBL)

Team-based learning (TBL) is a collaborative educational strategy frequently employed in medical education to promote student engagement, enhance knowledge retention, and develop critical thinking skills. Michaelsen et al. demonstrated that TBL effectively fosters critical thinking and facilitates the application of knowledge [10]. Similarly, Haidet et al. highlighted its role in improving student engagement and learning outcomes in medical training [11]. The three key phases in TBL, namely preparation, readiness assurance, and application, were implemented in this study.

Vertical integration

Vertical integration is a vital tool that facilitates early clinical exposure, enabling students to better understand the clinical applications of their learning. Harden was the pioneer in introducing vertical integration into curriculum planning [12]. While discussing medical education in the Netherlands, Ten Cate highlighted their transition from the H-shaped model to the Z-shaped model through vertical integration. With the adoption of competency-based medical education, the Indian system is also moving toward the Z-shaped model [13]. Vertical integration was very well planned and integrated in this study, which is evident from students' feedback. Brainstorming and reinforcing the concepts were also encouraged by the modified coffee cup model. Reinforcing the concepts by small group teaching was done to clarify the concept at the end of the activity. Pensak did an anatomical study on the 20 cavernous sinuses in patients, and his work likely contributes to the evolving understanding of this region's complex neurovascular relationships and the advancements in diagnostic and therapeutic techniques [14].

While studying the cavernous sinus has opened windows for many new surgical approaches, such as endonasal, transorbital, and transcranial, the structures inside the cavernous sinus, like the internal carotid artery, have been studied in detail [6,15]. The students were enthusiastic and happy about the whole exercise. They gave positive feedback regarding the exercise. Feedback responses have been summarized in Figures 2, 3, 4. A total of 78.8% of the students admitted that the exercise was helpful in understanding the complex anatomy of cavernous anatomy when questioned. When probed about their knowledge about cavernous sinus before, 79.8% of the students answered that they had some knowledge about the cavernous sinus, and 91.3% students answered that the activity helped in increasing their knowledge about cavernous sinus. When asked about the difficulties faced through the activity, the students mentioned about the time constraints and lack of more materials to make the model. A total of 94.2% of the students wanted more sessions like this in the future. When inquired about "the rate of the integration of neurosurgeon's lecture to explain the anatomical basis of cavernous sinus," 66.3% of students gave a score of 5, and 26% of students gave a score of 4 on Likert scale. Ninety-nine percent of the students wanted more clinically integrated classes. When asked about their experience, the students were very positive about the learning that happened during the activity, and some of the student responses were good, fantastic, fun with learning, and will never forget.

Limitations

The study involved pre-activity work that was strenuous. During the activity, the students felt that the time given to make the model was less. The materials were limited as there were 25 groups. Assessing the students after the activity was also strenuous.

## Conclusions

The modified coffee cup model is an innovative, simple, easy, versatile, and highly reproducible teaching method that enhances the understanding of the anatomy of the cavernous sinus. This approach introduces several key innovations, including learning by doing, team-based learning, and vertical integration. Notably, it is the first study of its kind in India.

## Appendices

**Table 3:** The individual scores of students for pre-test and post-test. Q: question

Q1		Q2		Q3		Q4		Q5		Q6		Q7		Q8		Q9		Q10		Q11		Q12	
Pre-test	Post-test	Pre-test	Post-test	Pre-test	Post-test	Pre-test	Post-test	Pre-test	Post-test	Pre-test	Post-test	Pre-test	Post-test	Pre-test	Post-test	Pre-test	Post-test	Pre-test	Post-test	Pre-test	Post-test	Pre-test	Post-test
5	5	5	5	5	5	5	5	0	5	0	5	5	5	0	5	0	5	5	5	5	5	5	5
5	5	5	5	5	5	5	5	5	5	5	5	5	5	5	5	5	5	5	5	5	5	0	0
5	5	5	5	5	5	5	5	0	0	0	5	5	5	5	5	5	5	0	5	5	5	0	5
5	5	5	5	5	5	5	5	0	0	5	5	0	5	5	5	5	5	5	5	5	5	5	5
0	5	5	5	5	5	0	5	0	5	5	5	5	5	0	5	0	5	0	0	0	5	0	0
5	5	5	5	5	5	5	5	0	5	0	5	5	5	5	5	5	5	5	5	5	5	0	0
5	5	5	5	5	5	5	5	5	5	5	5	5	5	5	5	5	5	5	5	5	5	5	5
5	5	0	5	5	5	5	5	0	5	0	5	0	5	0	5	5	5	0	5	5	5	0	5
5	5	0	0	5	5	5	5	5	5	5	5	5	5	5	5	5	5	0	0	5	5	5	5
5	5	0	5	5	5	5	5	5	0	5	5	0	0	5	5	0	0	0	0	0	0	5	5
5	5	0	0	5	5	5	5	0	5	0	5	5	0	5	5	5	5	5	5	0	0	5	5
5	5	0	5	5	5	5	5	0	0	5	5	0	0	5	5	0	0	0	0	5	5	5	5
5	5	5	5	5	5	5	5	5	5	5	5	5	5	5	5	0	5	5	5	5	5	5	5
5	5	5	5	5	5	5	5	0	0	5	5	5	5	5	5	5	5	5	5	5	5	5	5
5	5	5	5	5	5	5	5	0	0	5	5	5	5	5	5	5	5	5	5	5	5	5	5
5	5	5	5	5	5	5	5	5	5	0	5	5	5	5	5	5	5	5	5	5	0	5	5
5	5	5	5	5	5	0	0	5	5	5	5	0	5	5	5	5	5	5	5	5	5	5	5
5	5	5	5	5	5	5	5	0	0	5	5	5	5	5	5	5	5	5	5	5	5	5	5
5	5	5	5	5	5	5	5	0	0	5	5	5	5	5	5	5	5	5	5	5	5	5	5
5	5	5	5	5	5	5	5	5	0	5	5	5	5	5	5	5	5	5	5	5	5	5	5
5	5	5	5	5	5	5	5	5	0	5	5	0	5	5	5	5	5	5	5	5	5	5	5
5	5	5	5	5	5	5	5	5	5	5	5	5	5	5	5	5	5	0	5	5	5	0	5
5	5	0	5	5	5	0	5	0	0	5	5	5	5	5	5	5	5	5	5	5	5	0	0
5	5	5	5	5	5	0	5	0	5	5	5	5	5	5	5	0	5	5	0	0	5	0	0
5	5	5	5	5	5	5	5	0	0	5	5	5	5	0	5	5	5	5	5	5	5	5	5
5	5	5	5	5	5	5	5	0	0	5	5	5	5	5	5	5	5	0	0	0	5	0	0
5	5	5	5	5	5	5	5	0	5	5	5	5	5	5	5	5	5	5	5	5	5	0	0
5	5	5	5	5	5	5	5	5	0	0	5	5	5	5	5	5	5	0	0	5	5	0	5
5	5	5	5	5	5	5	5	0	0	0	5	5	5	5	5	5	5	5	5	0	5	5	5
5	5	5	5	5	5	5	5	5	0	0	5	0	5	5	5	5	5	5	5	0	5	0	0
5	5	0	5	5	5	0	5	0	5	0	5	5	5	5	5	5	5	5	5	5	5	5	0
5	5	5	5	5	5	5	5	0	0	5	5	5	5	5	5	0	0	5	5	0	0	0	0
5	5	5	5	5	5	5	5	5	0	5	5	5	5	5	5	5	5	5	5	5	5	5	5
5	5	5	5	5	5	5	5	0	0	5	5	0	0	5	5	5	5	5	5	5	5	5	5
5	5	5	5	5	5	5	5	0	0	5	5	5	5	0	5	5	5	0	5	5	5	0	5
5	5	5	5	5	5	5	5	0	5	5	5	5	5	5	5	5	5	0	5	5	5	5	5
5	5	5	5	5	5	5	5	0	0	0	5	5	5	5	5	0	5	5	5	0	5	5	5
5	5	5	5	5	5	5	5	5	5	5	5	5	5	5	5	5	5	0	5	5	5	5	5
5	5	5	5	5	5	5	5	5	5	0	5	5	5	5	5	5	5	5	5	5	5	5	5
5	5	5	5	5	5	5	5	5	5	5	5	5	5	5	5	0	5	5	5	5	5	5	0
5	5	5	5	5	5	5	5	0	5	5	5	5	5	5	5	5	5	0	5	5	5	5	5
5	5	5	5	5	5	5	5	5	5	5	5	5	5	5	5	0	0	5	5	5	5	5	5
5	5	0	5	5	5	5	5	0	0	0	5	5	5	0	5	5	5	5	5	5	5	5	0
5	5	5	5	5	5	5	5	0	0	0	5	5	5	5	5	5	5	5	5	5	5	5	5
5	5	5	5	5	5	5	5	5	5	5	5	5	5	5	5	5	5	5	5	5	5	5	5
5	5	5	5	5	5	5	5	5	5	5	5	5	5	5	5	5	5	5	5	5	5	5	5
5	5	5	5	5	5	5	5	5	5	5	5	5	5	5	5	5	5	5	5	5	5	5	5
0	5	5	5	0	5	5	5	5	5	5	5	5	5	5	5	5	5	0	5	5	5	5	5
5	5	0	0	5	5	5	5	5	5	5	5	5	5	5	5	5	5	5	5	5	5	0	5
5	5	0	5	5	5	5	5	0	5	5	5	0	5	5	5	5	5	5	5	5	5	0	5
5	5	5	5	5	5	5	5	0	0	5	5	5	5	5	5	5	5	5	5	5	5	5	5
5	5	5	5	5	5	5	5	0	5	5	5	5	5	5	5	5	5	5	5	5	5	5	5
5	5	5	5	5	5	5	5	5	5	5	5	5	5	5	5	5	5	5	5	5	5	5	5
5	5	5	5	5	5	5	5	5	5	0	5	5	5	5	5	5	5	5	5	5	5	0	0
0	0	0	5	5	5	5	5	0	5	0	5	5	5	5	5	5	5	5	5	5	5	5	5
5	5	5	5	5	5	5	5	5	5	5	5	5	5	5	5	5	5	5	5	5	5	5	5
5	5	5	5	5	5	5	5	5	5	5	5	5	5	5	5	5	5	5	5	5	5	5	5
5	5	5	5	5	5	5	5	5	5	5	5	5	5	5	5	5	5	5	5	5	5	5	5
5	5	0	5	5	5	5	5	5	5	5	5	5	5	5	5	0	5	5	5	5	5	5	5
5	5	5	5	5	5	5	5	0	0	0	5	5	5	5	5	5	5	5	5	5	5	5	5
5	5	5	5	5	5	5	5	0	0	0	5	5	5	5	5	5	5	5	5	0	5	0	5
5	5	5	5	5	5	5	5	5	5	5	5	5	5	5	5	5	5	5	0	0	5	0	5
5	5	0	0	5	5	5	5	0	5	0	5	5	5	0	0	5	5	5	5	5	5	5	5
5	5	5	5	5	5	5	5	5	0	5	5	5	5	5	5	5	5	5	5	5	5	0	5
5	5	5	0	0	5	5	5	0	0	0	5	5	5	5	5	5	5	0	0	5	5	5	5
5	5	0	0	5	5	5	5	5	5	5	5	5	5	5	5	5	5	0	0	5	5	0	5
5	5	5	5	5	5	5	5	5	5	5	5	5	5	5	5	0	0	5	5	5	5	5	5
5	5	0	0	5	5	5	0	0	0	0	5	0	0	5	5	5	5	5	5	0	5	5	0
5	5	5	5	5	5	0	5	0	0	0	5	5	5	5	5	0	5	5	5	5	5	0	5
5	5	5	0	5	5	5	5	0	0	5	5	0	0	5	5	0	0	5	5	5	5	0	0
5	5	5	5	5	5	5	5	5	0	0	5	5	5	5	5	5	5	5	5	5	5	0	5
5	5	0	0	5	5	5	5	5	5	5	5	5	5	5	5	5	5	5	5	0	5	5	5
5	5	5	5	5	5	5	5	5	5	5	5	5	5	5	5	5	5	0	0	5	5	5	5
5	5	5	5	5	5	5	5	5	5	5	5	5	5	5	5	5	5	5	5	5	5	5	5
5	5	5	5	5	5	5	5	0	0	0	5	0	0	5	5	0	0	5	5	0	5	0	0
5	5	5	5	5	5	5	5	5	5	5	5	5	5	0	0	5	5	5	5	5	5	5	5
5	5	5	5	5	5	0	0	5	5	5	5	0	0	5	5	5	5	5	5	5	5	5	5
5	5	5	5	5	5	5	5	5	5	5	5	5	5	5	5	5	5	5	5	5	5	0	5
5	5	5	5	5	5	5	5	0	5	5	5	5	5	5	5	5	5	5	5	5	5	0	5
5	5	5	5	5	5	5	0	5	0	5	5	5	5	5	5	5	5	5	5	5	5	5	5
5	5	5	5	5	5	5	5	0	0	0	5	5	5	5	5	5	5	0	0	5	5	0	0
5	5	5	5	5	5	5	5	5	5	5	5	5	5	5	5	5	5	5	5	0	0	5	5
5	5	5	5	5	5	5	5	0	0	5	5	5	5	5	5	5	5	5	5	5	5	5	5
5	5	5	5	5	5	5	5	0	5	0	5	5	5	5	5	5	5	5	5	5	5	5	5
5	5	5	5	5	5	5	5	0	5	5	5	5	5	0	5	5	5	5	5	5	5	5	5
5	5	5	0	5	5	5	5	0	0	5	5	5	5	5	5	0	5	5	5	5	5	0	0
5	5	5	5	5	5	5	5	5	0	5	5	5	5	5	5	5	5	5	5	0	5	0	0
5	5	5	5	5	5	5	5	0	0	5	5	5	5	5	5	5	5	5	5	0	5	5	5
5	5	5	5	0	5	5	5	0	5	0	5	5	5	5	5	5	5	5	5	5	5	0	5
5	5	0	0	5	5	5	5	5	5	0	5	5	5	5	5	5	5	5	5	5	5	5	5
5	5	5	5	5	5	5	5	0	5	5	5	5	5	5	5	5	5	5	5	5	5	0	0
5	5	5	5	5	5	5	5	0	5	5	5	5	5	5	5	5	5	5	5	5	5	0	0
0	5	5	0	0	5	5	5	5	5	5	5	5	5	0	0	0	0	0	5	5	5	5	0
4.784946	4.946237	4.086022	4.354839	4.784946	5	4.623656	4.784946	2.365591	2.903226	3.494623656	5	4.301075	4.569892	4.462366	4.83871	4.086022	4.569892	3.978495	4.354839	4.086022	4.731183	3.225806	3.817204
0.083246	-	0.166748	-	0.04489	-	0.259049	-	0.067619	-	1.03082E-08	-	0.058367	-	0.007453	-	0.002274	-	0.034068	-	0.001057	-	0.015555	-
